# Promising Anti-Biofilm Agents and Phagocytes Enhancers for the Treatment of *Candida albicans* Biofilm–Associated Infections

**DOI:** 10.3389/fcimb.2022.807218

**Published:** 2022-07-01

**Authors:** Yasmine H. Tartor, Gamal A. Elmowalid, Mohamed N. Hassan, Asmaa Shaker, Dalia F. Ashour, Taisir Saber

**Affiliations:** ^1^ Department of Microbiology, Faculty of Veterinary Medicine, Zagazig University, Zagazig, Egypt; ^2^ Department of Microbiology, Veterinary Hospital, Faculty of Veterinary Medicine, University of Sadat City, Sadat City, Egypt; ^3^ Department of Public Health, Dakahlia Veterinary Medicine Directorate, Mansoura, Egypt; ^4^ Department of Medical Microbiology and Immunology, Faculty of Medicine, Zagazig University, Zagazig, Egypt; ^5^ Department of Clinical Laboratory Sciences, College of Applied Medical Sciences, Taif University, Taif, Saudi Arabia

**Keywords:** *Candida albicans*, biofilm, phagocytes, fluconazole resistance, cinnamon oil, surfactants, antimicrobial proteins

## Abstract

Little is known about the interactions among phagocytes and antifungal agents and the antifungal immunomodulatory activities on *Candida* species biofilms. Here, inhibition of *C. albicans* biofilms and the interactions among biofilms and phagocytes alone or in combination with essential oils, biological, and chemical agents, or fluconazole were investigated. Biofilm formation by a panel of 28 C*. albicans* clinical isolates from hospitalized patients, birds, and cattle was tested. The anti-biofilm activities of cinnamon and clove oils, sodium dodecyl sulfate (SDS), cetyltrimethylammonium bromide (CTAB), and *Enterococcus faecalis* cell-free supernatant (CFS) in comparison with fluconazole were investigated using crystal violet and XTT reduction assays, expression of hypha-specific and hyphal regulator genes, and scanning electron microscopy (SEM) analysis. Of the tested *C. albicans* isolates, 15 of 28 (53.6%) were biofilm producers. Cinnamon followed by *E*. *faecalis*–CFS, SDS, and CTAB was the most effective inhibitors of planktonic *C. albicans* and biofilms. Fluconazole was an ineffective inhibitor of *C. albicans* biofilms. Sessile minimal inhibitory concentration (SMIC_50_) of cinnamon, SDS, CTAB, and *E. faecalis–*CFS downregulated the hypha-specific and regulator genes, albeit to various extents, when compared with untreated biofilms (*P* < 0.001). SEM analysis revealed disruption and deformity of three-dimensional structures in cinnamon oil–treated biofilms. *C. albicans* sessile cells within biofilm were less susceptible to phagocytosis than planktonic cells. The additive effects of phagocytes and the tested antifungals enabled phagocytes to engulf *C. albicans* cells rapidly in cinnamon, *E. faecalis*–CFS, or SDS-treated biofilms. No differences in anti-*Candida* or anti-biofilm eradication activities were detected among the tested isolates. Our findings reinforce the substantial anti-biofilm activity of cinnamon oil, SDS, and *E. faecalis–*CFS and provide new avenues for the development of novel anti-biofilm immunotherapies or antifungals that could be used prior to or during the management of cases with biofilm-associated infections.

## 1 Introduction

*Candida albicans* is the most prevalent fungal species of microbiota that colonizes the mucosal surfaces in the oral cavity, genitourinary tract, and gastrointestinal tract of humans, animals, and birds. However, as an opportunistic pathogenic fungus, it can exploit immune suppression, changes in resident microbiota, stress, and other factors to cause a wide range of infections, from superficial mucosal to life-threatening systemic candidiasis (Nobile and Johnson, 2015; Scaduto and Bennett, 2015). Of all the invasive fungal infections, candidiasis is by far the most common (Mayer et al., 2013). The ability of *C. albicans* to reversibly switch between yeast and filamentous form is a clinically significant virulence trait, rendering it the most commonly associated fungal species with biofilm formation (Ramage et al., 2005; Tsang et al., 2012; Pierce et al., 2017). The majority of mucosal and systemic candidiasis are associated with the formation of biofilm, a three-dimensional structure made up of adherent yeast cells, pseudohyphae, and hyphae enclosed in an extracellular polysaccharide matrix, on inert or biological surfaces (Ramage et al., 2001; Ghannoum et al., 2015). The sessile cells within a biofilm have unique characteristics from their free-floating cells (planktonic), the most notable of which are elevated resistance to antimicrobial drugs and immune responses, making *Candida* biofilm–associated infections a serious clinical issue that requires a multifaceted strategy for control (Nobile and Johnson, 2015). The dispersal of fungal components from the biofilm can also lead to disseminated host infection (Ghannoum et al., 2015). Antimicrobial coatings and medical device surface alterations provide intriguing possibilities for preventing biofilm formation on medical equipment (Percival et al., 2015).

Several studies investigating the activities of commercial antifungal drugs including fluconazole (FLC), itraconazole (ITC), ketoconazole (KTC), flucytosine, amphotericin B (AMB), and echinocandins against *Candida* biofilms have been carried out (Maiolo et al., 2014; Vila et al., 2016). However, these studies have found that increased resistance of sessile cells within biofilms to drugs is the most clinically relevant phenotypic alteration compared with their planktonic counterparts. Long-term therapy with antifungals inevitably results in evolving strains, with a subsequent increase in the prevalence of drug-resistant *C. albicans* (Judan Cruz et al., 2021). Hence, new and innovative antifungals are urgently needed to treat the recalcitrant *Candida* in biofilms. Essential oils (EOs) have been recognized to have anti-inflammatory, immunomodulatory, and antimicrobial activity against bacteria, fungi, and even SARs-CoV-2 virus (Chouhan et al., 2017; Tartor and Hassan, 2017; Asif et al., 2020; Mahboub and Tartor, 2020). The significant antifungal activity of EOs implies that they might be used as a natural anti-biofilm product (Sharifzadeh et al., 2016; El-Baz et al., 2021). Other less toxic natural products that have received attention are antimicrobial proteins. Interestingly, Cruz et al. (2013) observed inhibition of *C. albicans* filamentation by a compound secreted from *Enterococcus faecalis*. Shekh and Roy (2012) characterized a potential *E. faecalis* anti-*Candida* factor that might be utilized to treat candidiasis in immunocompromised patients. Surfactants, such as cetyltrimethylammonium bromide (CTAB) and sodium dodecyl sulfate (SDS), and amphiphilic chemicals are used in a variety of applications and can inhibit *C. albicans* growth and morphogenesis due to mitochondrial depolarization and abnormal organization of the actin skeleton, respectively (Yu et al., 2015).

The destruction of fungal cells by phagocytes of the innate immune system, such as polymorphonuclear leukocytes (PMNs) and macrophages, is an essential initial line of defence against *C. albicans* infections (Rudkin et al., 2013; Alonso et al., 2017). Interactions between phagocytes and planktonic *Candida* have been investigated in previous research (Lewis et al., 2012; Rudkin et al., 2013). Nevertheless, the response of *Candida* within established biofilms to phagocytes, and the interaction between antifungal agents and phagocytes against *Candida* biofilms has received little attention. One of the earlier studies in this direction is that of Katragkou et al. (2010), which looked at the interactions between phagocytes and *C. albicans* biofilms alone and in combination with antifungal drugs. The availability of such information is essential, as it might aid in the discovery of novel therapeutic anti-biofilm agents as well as the improved management of biofilm-associated infections.

The aims of this study were to ascertain the anti-*Candida* activities of natural (cinnamon and clove oils), chemical (CTAB and SDS), and biological *E. faecalis* cell-free supernatant (*E. faecalis*–CFS) compounds in comparison with FLC on *C. albicans* planktonic cells, adherent cells and subsequent biofilm formation, and preformed biofilms. In addition, the study sought to elaborate the additive anti-biofilm and the immunomodulatory effects of compounds on peripheral blood phagocytes.

## 2 Materials and Methods

### 2.1 *Candida albicans* Isolates

This study included 28 clinical *C. albicans* isolates and one reference strain from the American Type Culture Collection (ATCC^®^ 90028). The clinical isolates were isolated from hospitalized patients with vaginal candidiasis (n = 5), oral candidiasis, and onychomycosis (two from each). Sixteen isolates were isolated from chickens (n = 14) and turkeys (n = 2) with crop mycoses, and three isolates were isolated from cases of calf diarrhea. All isolates were identified based on macro- and micro-morphological, physiological, and biochemical characteristics as well as matrix-assisted laser desorption ionization time-of-flight mass spectrometry (VITEK MS, Biomerieux, Marcy I’Etoile, France). We examined macromorphology on HiCrome Candida differential agar medium (Himedia Laboratories, Mumbai, India) and micromorphology on rice agar with Tween 80, germ tube production, fermentation, and the assimilation of carbohydrates tests (Kurtzman and Fell JW, 2011). All isolates were maintained on Sabouraud dextrose agar slopes (Oxoid Ltd., Cambridge, UK) at 4°C.

The susceptibility of these isolates to antifungal drugs including AMB (100 I.U.), FLC (10 μg), KTC (10 μg), clotrimazole (CLT, 10 μg), ITC (10 μg), and nystatin (NYT, 100 I.U.) was determined following the Clinical and Laboratory Standards Institute CLSI M44 disc diffusion method (CLSI, 2020).

### 2.2 The Tested Essential Oils, Biological, and Chemical Agents

Clove and cinnamon EOs (Sigma Aldrich, St. Louis, MO, USA) were diluted in 1% dimethyl sulfoxide (DMSO, Sigma) (Shahzad et al., 2014). The CFS of *E. faecalis* used in this study was purified using ammonium sulfate precipitation and dialysis from two strains in our previous study (Hassan et al., 2018). These strains were isolated from cheddar cheese (CFS1) and chicken intestine (CFS2) samples. The surfactants CTAB and SDS were purchased from Sigma Aldrich and stock solutions prepared in sterile distilled water and then sterilized through 0.22-μm filters (Dusane et al., 2012). FLC (Pfizer, Inc., New York, NY) was dissolved in RPMI 1640 adjusted to pH 7.0 with 0.165 M morpholinepropanesulfonic acid (Sigma-Aldrich, St. Louis, Mo., USA) and used as a standard antifungal agent.

### 2.3 Detection of Biofilm Forming *C. albicans* Isolates

The isolates were tested for their ability to form biofilms using a 96-well microtiter plate-based method as described previously (Pierce et al., 2008; Dhanasekaran et al., 2014). Briefly, a loopful of colonies from fresh agar plates were cultured in yeast peptone dextrose medium (Oxoid Ltd., Cambridge, UK), overnight at 30°C at 150 rpm in an orbital shaker (Lab-line Incubator Shaker; Elliott Bay Laboratory Services Inc., Seattle, WA, USA). The yeast cells were harvested by centrifugation at 3,000 rpm for 10 min, washed twice in sterile phosphate-buffered saline (PBS, Sigma-Aldrich, St. Louis, Mo., USA), resuspended in RPMI 1640, counted using a hemocytometer, and concentration-adjusted to 1 × 10^6^ cells/ml. Aliquots of 100 µl of cell suspension were pipetted into wells of sterile, polystyrene, flat-bottom 96-well microtiter plate (Costar, Corning Inc., USA) and incubated at 37°C for 48 h. Broth free of cells was used as negative controls. Subsequently, the biofilm was washed three times with 200 μl of PBS to remove planktonic (free-floating) cells. The microtiter plate was drained by tapping and blotting with paper towels. The adherent biofilm layer on the plate was stained with 0.1% (w/v) crystal violet for 20 min and then fixed with 200 μl of 96% ethanol after rinsing off excess stain by washing with deionized water. The optical density (OD) value of the stained biofilm was measured with a micro-ELISA auto reader (Model 680, Bio Rad) at 492 nm. The isolates were classified into strong (OD > 0.320), moderate (OD 0.120–0.320), weak (OD < 0.120), and non-biofilm producers (OD ≤ OD of control). Each isolate was tested in triplicate on three separate occasions. Microscopic examination of biofilms was performed using an inverted microscope (Olympus, Japan).

### 2.4 Effect of the Tested Agents on *C. albicans* Planktonic Cells

The antifungal potential of the tested agents toward planktonic cells was determined by the broth microdilution test following CLSI M27-A3 standard (CLSI, 2008). Serial two-fold dilutions were performed in RPMI 1640 in each well to obtain concentration ranges from 1 to 1,024 μg/ml for FLC, 0.00048 to 1,024 μg/ml for clove and cinnamon EOs, and 0.25 to 1,024 μg/ml for *E. faecalis*–CFS. The tested concentrations of SDS and CTAB were 0.005 to 10% w/v. One hundred microliters of yeast cells (0.5 × 10^3^ CFU/ml) were added to each well. Positive (yeast cells grown in the absence of antifungal agents) and negative controls [RPMI 1640 containing DMSO 1% (v/v)] were included. After incubation for 48 h at 37°C, the lowest concentration of agent that reduced growth compared with that of the positive control was considered the planktonic minimum inhibitory concentration (PMIC). The test was performed in triplicate on three separate occasions.

### 2.5 Anti-Biofilm Activities of Compounds on *C. albicans* Biofilm Formation

The effects of different antifungal agents on hyphal growth and on the initial stages of biofilm formation (preventative) were evaluated after plating and incubation of a standard (1 × 10^6^ cells/ml) *C. albicans* suspension in 96-well, flat-bottom microtiter plates with agitation. At 1.5 h after adhesion, the non-adherent cells were removed by washing each well with PBS. The adherent cells were treated with 200 μl of various concentrations of the tested agents that had previously been serially diluted in a separate microtiter plate and were incubated for a further 24 h at 37°C under agitation. The crystal violet assay was used to quantify the resulting biofilm biomass compared with untreated controls, and the mitochondrial dehydrogenase activity of *C. albicans* biofilms was also determined using the 2,3-*bis*(2-methoxy-4-nitro-5-sulfo-phenyl)-2H-tetrazolium-5-caboxanilide (XTT) reduction assay (Tsang et al., 2012; Shahzad et al., 2014). Briefly, XTT-menadione solution: 40 µl of XTT (Sigma-Aldrich Corp.; 1 mg/ml prepared in PBS) and 2 μl of menadione (Sigma-Aldrich Corp.; 0.4 mM prepared in acetone) were mixed with PBS (158 μl), added to pre-washed biofilm adherent cells in each well and negative controls, and incubated in the dark for 3 h at 37°C. Subsequently, 100 μl of the solution was transferred to each well of new 96-well plates, and the OD was measured at 492 nm using a Labsystem Multiskan Ex microtiter plate reader. The colorimetric readings were subtracted from the values for negative controls (background XTT levels), and the metabolic activity (%) was calculated by the following equation: (mean OD_492_ of sample/mean OD_492_ of untreated control × 100). Sessile minimum inhibitory concentrations (SMICs) were considered at 50% (SMIC_50_) and 80% (SMIC_80_) reduction in metabolic activity in comparison to the untreated control biofilms (Pierce et al., 2008).

### 2.6 Effect of Agents on Preformed *C. albicans* Biofilms (Biofilm Destruction Testing)

After biofilm formation for 24 h at 37°C as described above, the medium was aspirated, and the biofilms were washed with PBS. A 200-μl volume of two-fold serial dilutions of agents in RPMI 1640 medium was added to each biofilm-containing well of the microtiter plates and incubated for 24 h at 37°C under agitation. The CV and XTT reduction assays were used for monitoring biofilm biomass and SMICs, respectively. All tests were performed on three independent occasions, each in triplicate.

### 2.7 Scanning Electron Microscopy of Biofilm Cells

The effect of the most active antifungal agent on morphology and biofilm structural integrity in comparison with control was examined by scanning electron microscopy (SEM) as previously described (Ramage et al., 2002b). *C. albicans* biofilms were formed on sterile plastic coverslips (15 mm in diameter; Nalge Nunc International) in 24-well tissue culture plate (Costar, Corning Inc., USA) by dispensing 2 ml of a cell suspension (1.0 × 10^6^ cells/ml) in RPMI 1640 for 24 h at 37°C. The cells were pretreated with SMIC_50_ of a test agent previously found to be highly effective in the earlier experiments. Discs containing biofilms were removed, washed with PBS, and placed in fixative [2.5% (v/v) glutaraldehyde in 0.1 M cacodylate buffer (pH 7.2)] overnight. After fixation, the cells were dehydrated with a series of ethanol washes, then immersed twice in hexamethyldisilizane, and air-dried in a desiccator. The samples were coated with gold (Baltec SDC 050 sputter coater) and examined with a Shimadzu Superscan SS-550 scanning electron microscope (Tokyo, Japan).

### 2.8 Reverse Transcriptase Real-Time PCR for Quantification Analysis of Biofilm-Associated Genes

Quantitative transcriptional analysis was used to study the effect of test agents on *C. albicans* adhesion (*ALS*3), filamentation (*HWP*1, *HYR*1), and the hyphal regulator *RAS*1 as previously described (Tsang et al., 2012). A 1-ml suspension of *C. albicans* cells was transferred into the wells of a pre-sterilized, flat-bottomed 24-well microtiter plates and incubated at 37°C for 1.5 h under agitation. Thereafter, the medium was aspirated followed by washing each well with PBS. Fresh RPMI 1640 medium (1 ml) containing SMIC_50_ of cinnamon oil, SDS, CTAB, FLC, and *E. faecalis*–CFS was added to each well and the plate was further incubated for 24 h at 37°C. Untreated control wells were included for comparison. After incubation, the wells were washed twice with PBS; then, buffer RLT (600 μl) was added to the wells; and sessile cells were scraped thoroughly, incubated for 10 min, and then transferred to 1.5-ml microcentrifuge tubes. Disruption was performed in a 2-min high-speed (30-Hz) tissue lyser for homogenization of samples. Total RNAs were then extracted using the RNeasy Mini Kit (Qiagen, Germany) following the manufacturer’s instructions. The real-time PCR mixture (25 µl) contained 2× QuantiTect SYBR Green PCR Master Mix (Invitrogen, Paisley, UK), 0.25 µl of RevertAid Reverse Transcriptase (Thermo Fisher), 0.5 µl of forward and reverse primers (20 pmol), 3 µl of template RNA, and 8.25 µl of nuclease-free water. Three independent replicates from each strain for each treatment were analyzed in triplicate using Stratagene MX3005P qPCR Systems (cycling conditions: 95°C for 20 s, 40 cycles of 95°C for 1 s and 60°C for 20 s) and Stratagene MX3005P software (Stratagene, Amsterdam, Netherlands). Gene expression was normalized to the *EFB*1 housekeeping gene according to the 2^−ΔΔCT^ method (Livak and Schmittgen, 2001).

### 2.9 Interactions Between Phagocytes and *C. albicans* Planktonic and Sessile Cells Alone or in Combination With Antifungal Agents

#### 2.9.1 Peripheral Blood Leucocyte Separation

Peripheral blood samples were collected from donors at health care settings and from cattle by specialists for separation of peripheral blood leucocytes (PBLs). The peripheral blood leucocyte cells (PBLCs) were separated from heparinized venous blood by dextran sedimentation and Ficoll centrifugation as previously described (Roilides et al., 1993). The cells were suspended in Hanks’ balanced salt solution, and the viability was determined after staining with trypan blue where cells with viability percent of less than 90 were excluded. Finally, the cells were counted on a hemocytometer, and their concentration was adjusted to 4 to 5 ×10^5^/ml in RPMI without serum to be used in the phagocytosis assay.

#### 2.9.2 Phagocytosis and Biofilm Eradication Assay

The assay was done using planktonic *C. albicans*, strong and moderate biofilm forming *C. albicans* isolates derived from hospitalized patients and birds’ crop mycoses and from cases of calf diarrhea. To test the PBLCs biofilm formation inhibitory and destructive activities, the PBLCs containing the phagocytic cells were added to strong and/or moderate biofilm forming *C. albicans* isolates from different sources and at different developmental phases or to planktonic *C. albicans* cultured in six-well cell culture plates. PBLCs (4 to 5 ×10^5^ viable cells/ml) were added to six-well cell culture plates (4 ml per well) containing planktonic cells, 48-h mature biofilms, and biofilms formed after treatment with SMIC_50_ of the tested antifungal agents (in biofilm inhibition and biofilm destruction testing as described in the aforementioned sections). The PBLCs were mixed gently and tapped to ensure even distribution of cells over the biofilm. Subsequently, the plates were incubated at 37°C for 15-, 30-, 60-, and 90-min intervals to let the phagocytic cells interact with the biofilm. Planktonic cells and antifungal untreated biofilms served as controls. At the determined time intervals, the phagocytes’ ability to destroy and clear biofilm was estimated under a high power of light microscope (Olympus Corporation, Tokyo, Japan), and photomicrographs were taken.

### 2.10 Data Analysis

The experiments were carried out in triplicate, and the results were presented as mean values with standard deviations (SD). The differences between means related to the effects of different antifungal agents (cinnamon, FLC, SDS, CTAB, and *E. faecalis*–CFS) on *C. albicans* biofilm were investigated by orthogonal comparisons according to Proc GLN (SAS, 2012). The relationships between the results of the XTT reduction assay and those obtained by the crystal violet method were determined by person correlation analysis (Proc CORR). Variance, homogeneity, and normality were examined by the Shapiro–Wilk and Levene’s tests. The level of statistical significance was set at *p*-value < 0.05.

## 3 Results

### 3.1 Antifungal Susceptibility and Biofilm Formation Ability of *C. albicans* Isolates

AMB exhibited antifungal activity against the tested *C. albicans* clinical isolates, with the overall rate of sensitivity being 96.4%. The highest resistance rate was observed to NYT (67.9%) followed by ITC (64.3%), CLT (50%), FLC (46.4%), and KTC (39.3%). The mean of the inhibition zone diameters ± SD was 20.79 ± 04.77 mm for AMB, CLT (18.39 ± 08.71 mm), ITC (11.64 ± 07.80 mm), FLC (15.86 ± 13.90 mm), KTC (29.27 ± 11.16 mm), and NYT (15.14 ± 4.46 mm). Two *C. albicans* isolates, from cases of vaginitis and onychomycosis, were susceptible to the six antifungal drugs tested (**Table 1**).

**Table 1:** Biofilm formation ability and resistance patterns of *C. albicans* isolates.

Isolate No.	Clinical Condition/Host	Resistance Pattern	OD at 492 nm	Biofilm Formation
1	Crop mycoses/chicken	FLC, ITC, KTC, and NYT	0.33 ± 0.01	Strong
2	Crop mycoses/chicken	ITC	0.086 ± 0.03	None
3	Crop mycoses/chicken	NYT	0.075 ± 0.10	None
4	Crop mycoses/chicken	CLT, ITC, and NYT	0.22 ± 0.05	Moderate
5	Crop mycoses/chicken	FLC and KTC	0.115 ± 0.01	Weak
6	Crop mycoses/chicken	CLT and ITC	0.072 ± 0.09	None
7	Crop mycoses/chicken	ITC, KTC, and NYT	0.07 ± 0.03	None
8	Crop mycoses/chicken	FLC, ITC, CLT, and NYT	0.13 ± 0.04	Moderate
9	Crop mycoses/chicken	FLC, ITC, CLT, KTC, and NYT	0.11 ± 0.02	Weak
10	Crop mycoses/chicken	FLC, CLT, and NYT	0.13 ± 0.07	Moderate
11	Crop mycoses/chicken	FLC, CLT, ITC, KTC, and NYT	0.32 ± 0.05	Strong
12	Crop mycoses/chicken	FLC, ITC, KTC, and NYT	0.09 ± 0.04	None
13	Crop mycoses/chicken	FLC, CLT, and NYT	0.08 ± 0.02	None
14	Crop mycoses/chicken	CLT and NYT	0.125 ± 0.01	Moderate
15	Crop mycoses/Turkey	CLT, ITC, KTC, and NYT	0.2 ± 0.10	Moderate
16	Crop mycoses/Turkey	CLT, ITC, and KTC	0.135 ± 0.01	Moderate
17	Thrush/human	ITC and NYT	0.08 ± 0.04	None
18	Thrush/human	NYT	0.07 ± 0.05	None
19	Vaginitis/human	ITC and NYT	0.11 ± 0.02	Weak
20	Vaginitis/human	AMB, FLC, CLT, KTC, and NYT	0.35 ± 0.09	Strong
21	Vaginitis/human	FLC, CLT, ITC, KTC, and NYT	0.135 ± 0.10	Moderate
22	Vaginitis/human	ND	0.096 ± 0.06	None
23	Vaginitis/human	ITC	0.095 ± 0.02	None
24	Onychomycosis/human	FLC, ITC, and NYT	0.091 ± 0.06	None
25	Onychomycosis/human	ND	0.075 ± 0.03	None
26	Diarrhea/calf	FLC and ITC	0.35 ± 0.04	Strong
27	Diarrhea/calf	FLC	0.088 ± 0.07	None
28	Diarrhea/calf	FLC, ITC, KTC, and NYT	0.2 ± 0.01	Moderate
*C. albicans* ATCC90028	Reference strain	CLT and NYT	0.12 ± 0.03	Moderate

AMB, amphotericin B; FLC, fluconazole; ITC, itraconazole; CLT, clotrimazole; KTC, ketoconazole; NYT, nystatin; ND, not detected (i.e., the isolate was sensitive to the tested antifungal drugs). Optical density (OD) value was measured using a micro-ELISA auto reader (Model 680, Bio Rad) at 492 nm. Values are expressed as mean ± standard deviations.

As depicted in **Table 1**, regardless of the isolate source, only some *C. albicans* isolates were able to form biofilm with different densities. Four *C. albicans* isolates (14.3%) were able to form strong biofilms, eight isolates (28.6%) were moderate biofilm producers, three isolates (10.7%) were weak biofilm producers, and 13 isolates (46.4%) were non-biofilm producers. *C. albicans* ATCC90028 reference strain formed a moderate biofilm. Biofilm forming isolates were resistant to at least two to five antifungals.

**Supplementary Figure 1** presents the stages of biofilm formation as follows: the early stage starts from 0 to 6 h and shows adherence and development of blastospores into distinct microcolonies predominantly of budding yeast cells (2–4 h). After 6–8 h, the appearance of germ tubes, pseudohyphae, and young hyphae within an extracellular matrix was detected. After that, the biofilm is said to be composed of a thick extracellular polysaccharide layer in which cells, pseudohyphae, and hyphae are embedded.

### 3.2 Activity of Antifungal Agents on Planktonic *C. albicans*, Biofilm Formation, and Preformed Biofilms

MIC values of clove and cinnamon oils, two chemical surfactants (CTAB and SDS), *E. faecalis–*CFS, and FLC on planktonic cells using the broth microdilution method are summarized in **Table 2**. The lowest MICs were observed for cinnamon oil (0.00048 to 0.03 µg/ml) and SDS (0.038 to 0.62%). Clove oil (0.25 to 2 µg/ml) and CTAB (0.15% to 2.5%) revealed higher MIC values than cinnamon oil and SDS, respectively. *C. albicans* planktonic cells were susceptible to *E. faecalis–*CFS1 (0.125 to 1 µg/ml) and CFS2 (0.25 to 1 µg/ml), whereas higher MIC values were observed for FLC (4 to 256 µg/ml).

**Table 2:** Minimum inhibitory concentration (µg/ml) values of different antifungals on planktonic *C. albicans*, biofilm formation, and preformed biofilm.

Antifungal Agent	Code of Test Strain	PMIC	Biofilm Formation		Preformed Biofilm	
			SMIC_50_	SMIC_80_	SMIC_50_	SMIC_80_
Fluconazole	**1**	256 ± 0.14	512 ± 1.75	1,024 ± 6.35	>1,024	>1,024
	**11**	128 ± 2.15	1,024 ± 5.93	>1,024	>1,024	>1,024
	**20**	128 ± 1.067	1,024 ± 4.1	>1,024	>1,024	>1,024
	**26**	128 ± 3.52	1,024 ± 5.2	>1,024	>1,024	>1,024
	15	8 ± 0.821	64 ± 1.42	256 ± 2.18	512 ± 2.415	>1,024
	16	4 ± 0.129	32 ± 0.742	128 ± 1.55	256 ± 2.935	>1,024
Cinnamon oil	**1**	0.015 ± 0.15	1 ± 0.21	2 ± 0.112	4 ± 0.521	4 ± 0.65
	**11**	0.0009 ± 0.18	0.5 ± 0.031	1 ± 0.271	2 ± 0.45	4 ± 0.18
	**20**	0.0019 ± 0.09	1 ± 0.21	2 ± 1.55	4 ± 2.48	8 ± 3.8
	**26**	0.03 ± 0.01	2 ± 0.42	4 ± 0.27	8 ± 0.58	8 ± 0.64
	15	0.00048 ± 0.0	0.5 ± 0.51	1 ± 0.01	2 ± 1.33	4 ± 2.6
	16	0.0009 ± 0.06	0.25 ± 0.034	0.5 ± 0.13	1 ± 0.05	2 ± 0.16
Clove oil	**1**	0.5 ± 0.346	1 ± 0.06	2 ± 0.97	4 ± 0.91	8 ± 2.13
	**11**	2 ± 0.64	4 ± 1.3	8 ± 0.19	16 ± 1.21	32 ± 2.67
	**20**	1 ± 0.18	2 ± 0.66	4 ± 0.83	8 ± 1.084	16 ± 1.13
	**26**	2 ± 0.58	4 ± 0.213	8 ± 0.954	16 ± 1.01	32 ± 1.72
	15	0.5 ± 0.157	1 ± 0.07	2 ± 0.14	4 ± 1.6	8 ± 1.55
	16	0.25 ± 0.144	0.5 ± 0.194	1 ± 0.031	2 ± 0.92	4 ± 0.58
SDS	**1**	0.62% ± 0.12	2.5% ± 0.28	2.5% ± 0.23	5% ± 0.63	10% ± 1.42
	**11**	0.31 ± 0.04	0.62 ± 0.18	1.25 ± 0.23	2.5 ± 0.37	5 ± 1.12
	**20**	0.31 ± 0.14	0.62 ± 0.57	1.25 ± 0.21	2.5 ± 0.34	5 ± 0.61
	**26**	0.15 ± 0.06	0.62 ± 0.11	0.62 ± 0.14	1.25 ± 0.27	2.5 ± 1.17
	15	0.019 ± 0.02	0.31 ± 0.25	0.31 ± 0.05	0.62 ± 0.36	1.25 ± 0.92
	16	0.038 ± 0.01	0.31 ± 0.42	0.62 ± 0.78	1.25 ± 0.91	2.5 ± 0.35
CTAB	**11**	1.25 ± 0.82	2.5 ± 1.72	2.5 ± 2.01	>10	>10
	**20**	1.25 ± 0.64	1.25 ± 1.14	1.25 ± 1.62	>10	>10
	**26**	0.62 ± 0.02	0.62 ± 0.52	1.25 ± 0.93	>10	>10
	15	0.15 ± 0.04	0.31 ± 0.07	0.62 ± 0.18	5 ± 1.34	10 ± 3.34
	16	0.31 ± 0.05	0.62 ± 0.19	1.25 ± 0.74	5 ± 1.5	10 ± 2.52
CFS1	**1**	0.5 ± 0.12	1 ± 0.23	2 ± 1.15	2 ± 1.56	4 ± 2.01
	**11**	1 ± 0.05	2 ± 0.11	4 ± 1.15	4 ± 1.96	8 ± 2.2
	**20**	0.5 ± 0.15	1 ± 0.24	1 ± 0.01	2 ± 0.19	4 ± 1.14
	**26**	1 ± 0.04	2 ± 1.01	4 ± 1.99	4 ± 2.1	8 ± 2.95
	15	0.25 ± 0.01	0.5 ± 0.08	1 ± 0.11	2 ± 1.74	2 ± 1.98
	16	0.125 ± 0.01	0.25 ± 0.09	0.5 ± 0.24	1 ± 1.07	2 ± 1.54
CFS2	**1**	1 ± 0.00	1 ± 0.09	2 ± 0.18	4 ± 0.94	8 ± 2.12
	**11**	0.25 ± 0.05	0.5 ± 0.12	1 ± 0.24	1 ± 1.09	2 ± 1.95
	**20**	1 ± 0.19	1 ± 1.1	2 ± 1.93	4 ± 2.43	8 ± 3.26
	**26**	0.5 ± 0.16	1 ± 1.24	2 ± 2.01	4 ± 2.71	8 ± 3.03
	15	0.5 ± 0.12	1 ± 1.4	1 ± 1.23	2 ± 2.14	4 ± 2.9
	16	0.25 ± 0.00	0.5 ± 0.15	1 ± 0.21	2 ± 1.91	2 ± 2.13

PMIC, planktonic MIC; SMIC50 and SMIC80, sessile MIC that inhibit 50% and 80% of biofilm metabolic activity that were determined using XTT reduction assay; CFS1, CFS2 cell-free supernatant of E. faecalis isolated from cheese and chicken. Isolates in boldface are strong biofilm producers whereas non-bold ones are moderate biofilm producers.

SMICs obtained by XTT assay for the tested antifungal agents against *C. albicans* of different biofilm densities are presented in **Table 2**. Each compound was tested alone to determine the individual antifungal activity. FLC was the least active against *C. albicans* biofilms (the most repeated SMIC was >1,024 µg/ml). The highest antifungal and anti-biofilm activities were obtained by cinnamon oil (for inhibition of biofilm formation, SMIC_50_ 0.25 to 2 µg/ml, SMIC_80_ 0.5 to 4 µg/ml; preformed biofilm, SMIC_50_ 1 to 8 µg/ml; and SMIC_80_ 2 to 8 µg/ml). Higher SMICs were observed for clove oil (biofilm formation, SMIC_50_ 0.5 to 4 µg/ml, SMIC_80_ 1 to 8 µg/ml; preformed biofilm, SMIC_50_ 2 to 16 µg/ml, SMIC_80_ 4 to 32 µg/ml). Biofilm formation of *C. albicans* was inhibited by 50% at a concentration of 0.31% to 2.5% of both SDS and CTAB, whereas 80% inhibition was obtained at 0.62% to 2.5% of SDS and 0.62% to 5% of CTAB. For inhibition of preformed biofilm, SMIC_50_ was 0.62% to 5% for SDS and 5% to >10% for CTAB, and SMIC_80_ was 1.25% to 10% and >10%. Interestingly, *E. faecalis–*CFS1 of 0.5 to 4 µg/ml was required to inhibit 80% of *C. albicans* biofilm formation. Nonetheless, *E. faecalis–*CFS1 and –CFS2 both of 1 to 4 µg/ml and 2 to 8 µg/ml reduced the viability of preformed biofilms by 50% and 80%, respectively.

Person correlation analysis showed a highly significant (*P* < 0.001) positive relationship between the results of the XTT reduction and crystal violet assays, regardless of the isolate source (*r* = 0.994, 0.997, 0.818, and 0.835 for isolates code nos. 1, 15, 20, and 26, respectively) in inhibition of biofilm formation. Moreover, in destruction of 24-h preformed biofilm testing, *r* = 0.979, 0.964, 0.712, and 0.801 for the same isolates, respectively. Overall, the XTT reduction assay was more reliable than the crystal violet assay for investigating the effect of antifungal agents on biofilm formation and destruction.

### 3.3 SEM Analysis for Visualization of *C. albicans* Biofilm and the Effect of Cinnamon Oil on Biofilm Architecture

**Figure 1:**
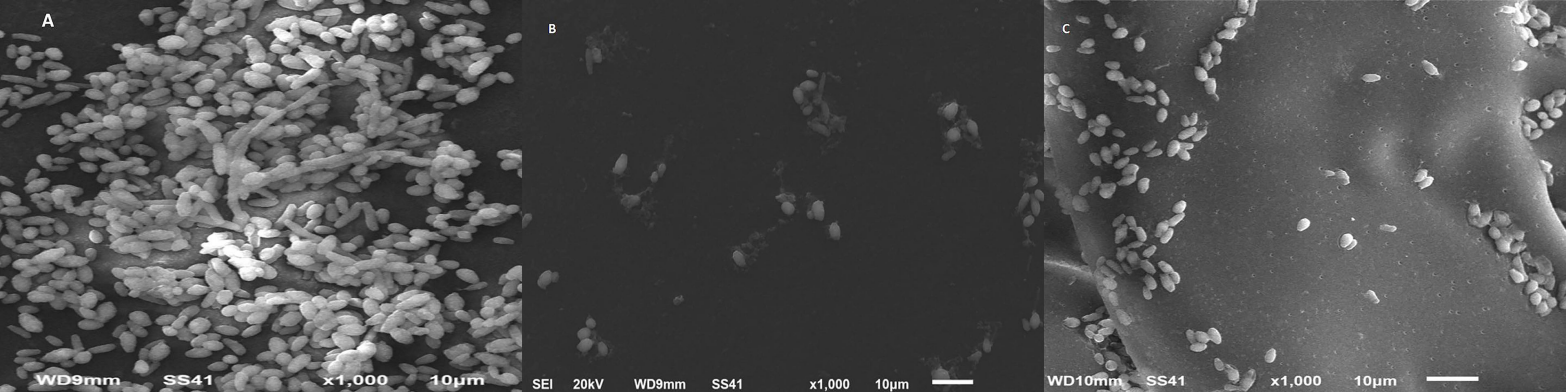
Scanning electron microscopy images of *C. albicans* biofilms. Cinnamon exhibited a profound inhibitory effect on *C. albicans* biofilm formation and preformed biofilms. **(A)** Mature biofilm with a multilayer of blastoconidia, pseudohyphae, and extracellular matrix. **(B)** Biofilm formation with cinnamon (1 µg/ml) showing small shrinkage blastoconidia and absence of pseudohyphae. **(C)** Twenty-four–hour preformed biofilm with cinnamon (4 µg/ml) showing a monolayer of small round blastoconidia.

The disruption and ultrastructural changes of *C. albicans* biofilms were examined by SEM. The disruption and ultrastructural changes of *C. albicans* biofilms in the presence of SMIC_50_ of cinnamon, the most active antifungal agent of the tested compounds, were examined by SEM. *C. albicans* biofilm architecture was changed in the presence of cinnamon oil. The cinnamon-free control biofilm consisted of a dense and heterogeneous network of yeast, pseudohyphae, and hyphae (**Figure 1A** **)**. The obtained results indicated that cinnamon of 1 µg/ml was sufficient to abrogate filamentation, resulting in atypical biofilm architecture consisting of a single layer of loosening yeast cells with significant changes in the morphology (**Figure 1B**). Cinnamon-treated (4 µg/ml) preformed biofilm exhibited disorganization of biofilm stages and the biofilm grew entirely as yeast cells and pseudohyphae. True hyphae were rarely observed, a factor that contributed to the poor biofilm architecture (**Figure 1C**).

### 3.4 Real-Time RT-PCR for Gene Expression Analysis

**Figure 2:**
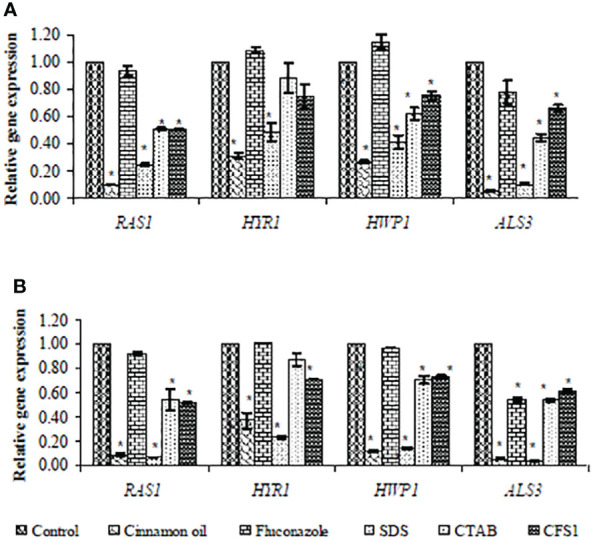
Fold change in expression of hypha-specific genes (*HYR1*, *HWP1*, and *ALS3*) and the hyphal regulator *RAS1* in strong biofilm producers *C. albicans* isolates (code nos. 20 **(A)** and 26 **(B)**, **Table 2**) after treatment with the sessile minimal inhibitory concentration (SMIC50) of cinnamon (1, 4 µg/ml), fluconazole (1,024 µg/ml), SDS (0.62%), CTAB (1.25, 0.62%), and CFS1 (1, 2 µg/ml), respectively. The results were the average of three independent experiments ± SD normalized to the housekeeping gene (*EFB1*) and relative to the untreated control isolate. * *P*< 0.001 when compared with the respective control.

SYBR green real-time RT-PCR was performed to assess expression of hypha-specific genes (*HYR*1, *HWP*1, and *ALS*3) and the hyphal regulator *RAS*1 in *C. albicans* biofilm with different antifungal agents. As presented in **Figure 2**, SMIC_50_ of cinnamon oil, SDS, CTAB, and *E. faecalis–*CFS showed significant (*p* < 0.001) downregulated expression of the hypha-specific and regulator genes of two strong biofilm producer isolates (code nos. 20 and 26), albeit to various extents, when compared with their respective untreated controls.

### 3.5 The Interaction Between Peripheral Blood Leucocytes and *C. albicans* Biofilms Alone and in Combination With Antifungals

**Figure 3:**
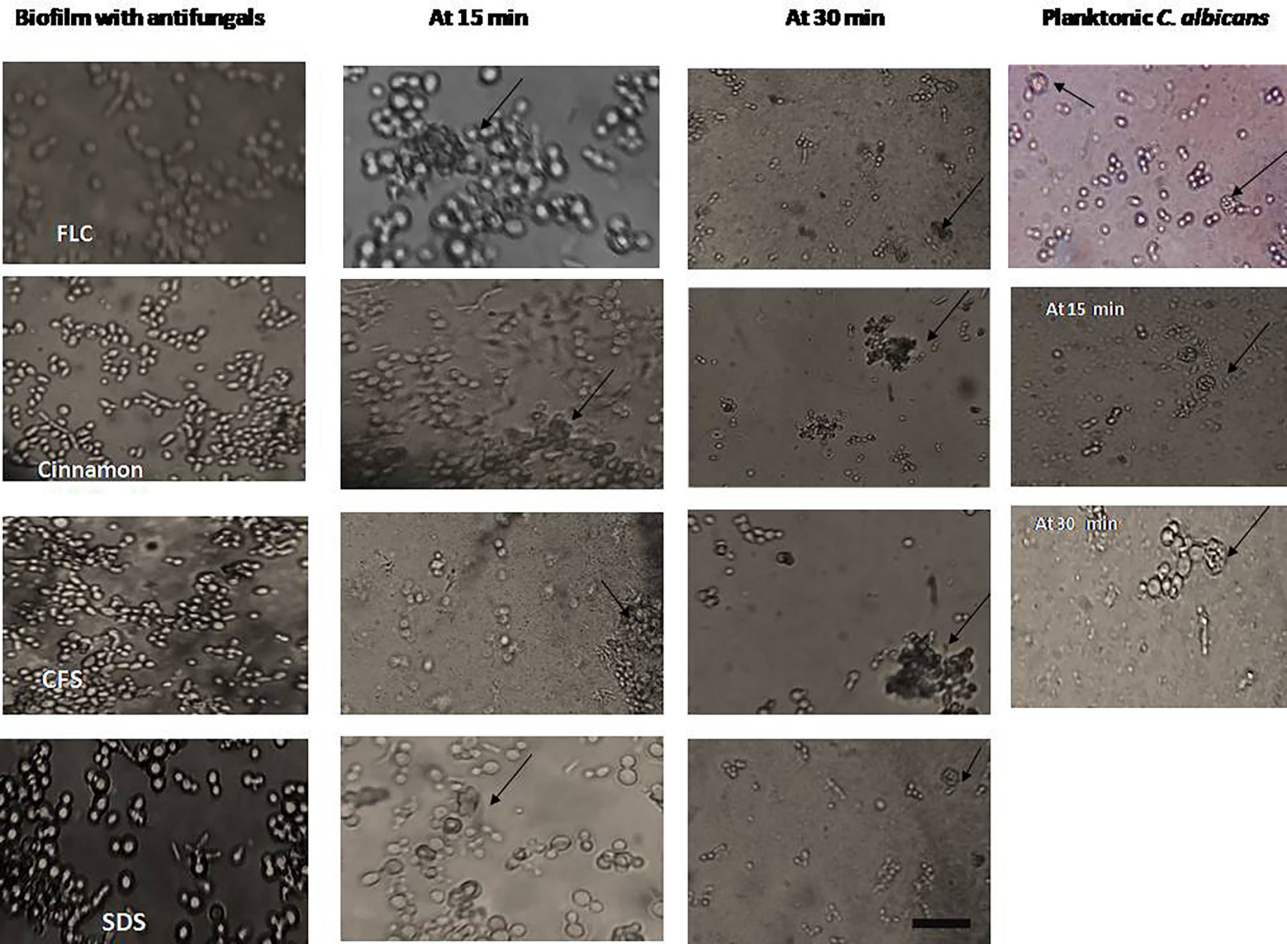
Interaction between peripheral blood leukocytes (PBLCs) and *C. albicans* biofilms formed in the presence of the tested agents (in biofilm inhibition testing): fluconazole, cinnamon oil, *E. faecalis*–CFS, and sodium dodecyl sulfate (SDS). Fluconazole (1,024 µg/ml)–treated biofilm was resistant to phagocytic cells. Meanwhile, in cinnamon (1 µg/ml)–, *E. faecalis*–CFS (1 µg/ml)–, and SDS (0.62%)–treated biofilms, the phagocytic cells were able to clean and engulf *Candida* cells. The phagocytic cells showed a considerable activity against *C. albicans* biofilm formation in the presence of *E. faecalis*–CFS (×400, scale bar = 40 µm). In another set of experiments PBLCs were added to planktonic cells. Both *C. albicans* biofilm with antifungals and planktonic cells were kept as controls. Arrows refer to PBLCs.

**Figure 4:**
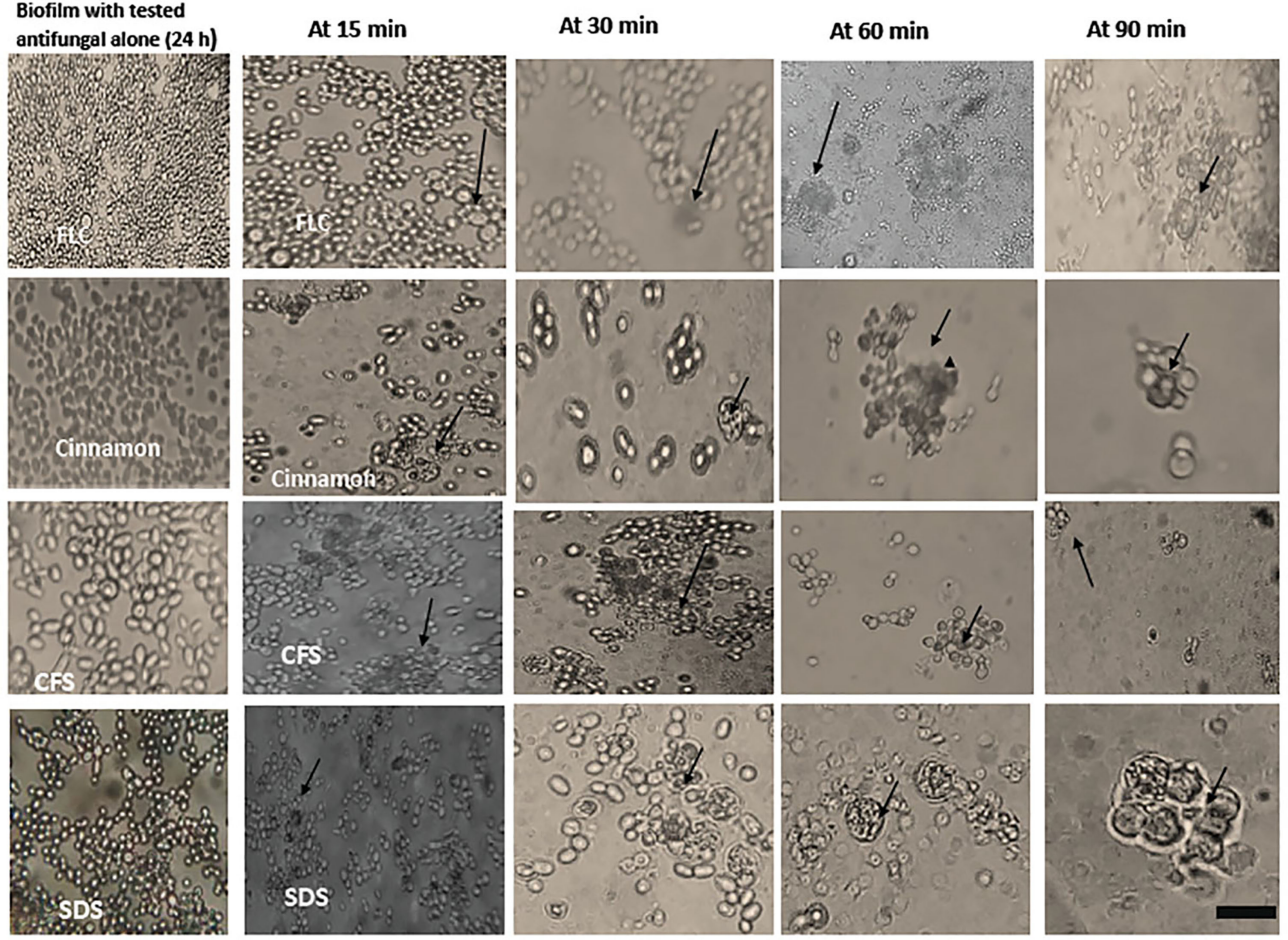
Interaction between peripheral blood leukocytes (PBLCs) and *C. albicans* biofilms alone or in combination with fluconazole (1,024 µg/ml), cinnamon oil (4 µg/ml), *E. faecalis*–CFS (2 µg/ml), and sodium dodecyl sulfate (SDS; 2.5%) at different time intervals. Biofilm treated with cinnamon, *E. faecalis*–CSF, and SDS were effectively cleared within 60 min of PBLCs addition. Meanwhile, the cells were not eradicated after 90 min in fluconazole-treated biofilm (×400, scale bar = 40 µm). Arrows refer to PBLCs.

**Figure 5:**
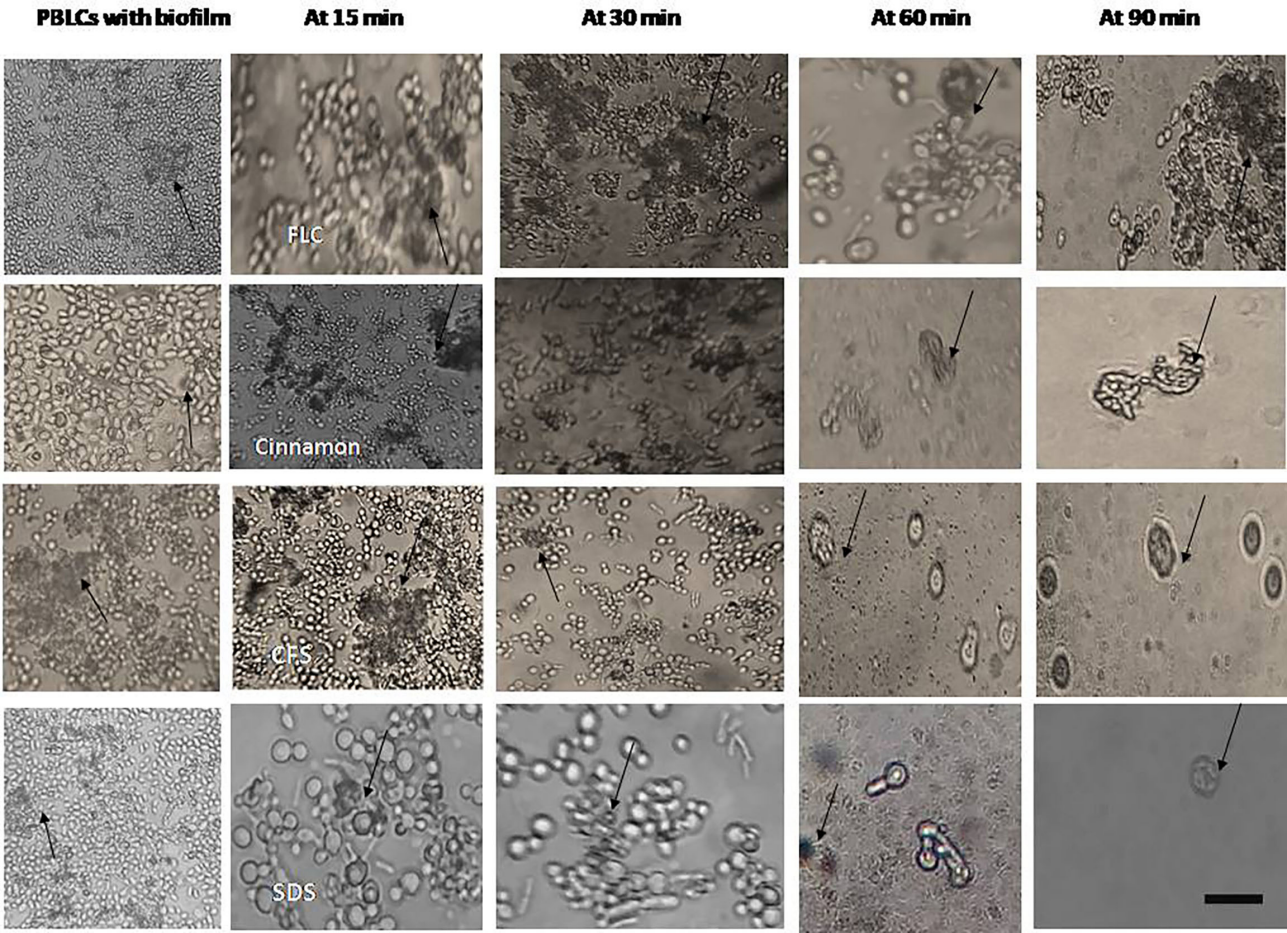
Interaction between peripheral blood leukocytes (PBLCs) and 24-h preformed biofilms treated with the tested agents (in biofilm destruction testing): fluconazole (1,024 µg/ml), cinnamon oil (4 µg/ml), *E. faecalis*–CFS (2 µg/ml), and sodium dodecyl sulfate (SDS, 2.5%). Additive effects between phagocytes and antifungal agents were observed. Biofilm clearance was detected at 15–90 min after PBLCs addition. Biofilms treated with cinnamon, *E. faecalis*–CSF, and SDS were effectively cleared within 60 min of PBLCs addition (×400, scale bar = 40 µm). Arrows refer to PBLCs.

Phagocytic cells were able to engulf *C. albicans* planktonic cell counterparts’ rapidly and effectively within 15–30 min after PBLC addition (**Figure 3**). Biofilms formed in the presence of the tested antifungals (inhibitory, during biofilm formation) were less confluent and were susceptible to phagocytic cells in the PBLC suspension. Phagocytic cell enlargement and aggregation were observed after 15 and 30 min in cinnamon and *E. faecalis*–CFS. The biofilms eradicated after 30 min of the PBLC addition in all biofilms pretreated with antifungal (**Figure 3**). PBLCs were also able to efficiently destroy *C. albicans* cells within 60 min in antifungal-treated mature biofilms and 24 h preformed biofilms-treated with the SMICs of the tested antifungals agents (in biofilm destruction testing) at different intervals (**Figures 4**, **5** **)** with predominance of cell aggregates and size enlargement. The combination of PBLCs and SMICs of cinnamon oil, FLC, SDS, and *E. faecalis–*CFS at various intervals revealed variation in the destruction of *C. albicans* biofilms.

## 4 Discussion

The increase in resistance to existing antifungals is a great stimulus to try novel therapeutic drugs of different origins. Therefore, it is crucial to explore alternative strategies to overcome the limitations of current therapeutic agents against biofilm associated fungal infection. This study investigated the antifungal activity of two herbal extracts (clove and cinnamon EOs), two chemical surfactants (CTAB and SDS), and an antimicrobial protein (*E. faecalis–*CFS) on *C. albicans* planktonic and biofilm growth as well as the interaction between PMNs and *C. albicans* biofilms alone and in combination with the tested antifungals in comparison with FLC. Antifungal susceptibility testing of *C. albicans* clinical isolates revealed that 14 isolates (50%) were resistant to two or three azoles. Our finding is in agreement with previous observations proposing that resistance of *C. albicans* isolates to azoles is a growing trend (Liu et al., 2014; Whaley et al., 2017). Similar to previous findings, AMB showed potent activity against *C. albicans* isolates (Dagi et al., 2016).

One of the major factors contributing to the virulence of *C. albicans* is its ability to acclimatize to a variety of different habitats for growth and the formation of biofilm. Several authors further stated that morphological transition allows *C. albicans* to adapt to different niches in all hosts (Scaduto and Bennett, 2015). In this study, 53.6% of isolates were biofilm positive by the microtiter plate method; 14.3% were strong biofilm producers, 28.6% were moderate biofilm formers, 10.7% were weak biofilm producers, and 46.4% were non-producers. This may reflect similarities in the ability of *C. albicans* isolates from different infection sites to form biofilms and strengthen the reports from other researchers that most *Candida* infections are associated with biofilm forming ability (Ramage et al., 2005; Tsang et al., 2012; Pierce et al., 2017). The obtained results were higher than those obtained by El-Baz et al. (2021) who found that 40.4% of *C. albicans* isolates were biofilm producers, whereas Khan et al. (2014) found that 88% of *C. albicans* clinical isolates were biofilm producers. The obtained results revealed that planktonic *C. albicans* cells were more susceptible to the tested antifungals than *C. albicans* biofilm (**Table 2**). This agrees with other findings that biofilms become less susceptible to antifungal treatment than their planktonic counterparts and is explained by several mechanisms, including the increased cell density inside the biofilm, extracellular matrix that acts as a drug sequestrant, and drug efflux pumps (Ramage et al., 2002a; Pierce et al., 2017). The varying levels of attenuation of biofilm formation by *Candida* cells in the presence of the tested antifungal agents were in a dose-dependent manner. Data obtained showed cinnamon oil and FLC MICs ranged from 0.00048 to 0.03 and 4 to 256 µg/ml for planktonic cells, SMIC_80_ 2 to 8 µg/ml, >1,024 µg/ml for preformed biofilm and 0.5 to 4 and 128 to >1,024 µg/ml for inhibition of biofilm formation, respectively. Serra et al. (2018) reported that MICs of cinnamon oil against *C. albicans* planktonic and biofilm growth were 0.03% and 0.01%. Szweda et al. (2015) reported a higher antifungal activity of cinnamon oil with MICs of 0.0006%–0.0096%, which confirms the high fungicidal activity of cinnamon oil. In addition, the safe and non-toxic nature of cinnamon oil to human and animal cells may have clinical relevance for treating candidiasis. The difference in antifungal resistance appears to be phase-specific, dependent on the stage of biofilm formation, and relies on the planktonic cell up to the mature biofilm. A variation in resistance was observed in this investigation (**Table 2**). This may be attributed to the changes in the level of ergosterol in the plasma membrane during biofilm formation stages. In the early stages of biofilm, the sterol level is the same as that present in planktonic cells, so sterol is an effective target for drug therapy during the early stages. Biofilms develop in parallel with decreased ergosterol levels over time, and their reliance on ergosterol decreases, potentially limiting the efficacy of antifungal drugs that target ergosterol (Mukherjee et al., 2003). However, the observations of this study lead to the suggestion that the tested antifungal agents such as oils target cell membranes in both the planktonic and sessile cells of *C. albicans* (Khan et al., 2014; Nazzaro et al., 2017).

SDS was found to be effective in disrupting *C. albicans* biofilms compared with CTAB. This goes hand in hand with previous researchers’ finding that SDS was more effective as an antifungal agent compared with rhamnolipids and CTAB toward *Yarrowia lipolytica* (Dusane et al., 2012). Vieira and Carmona-Ribeiro (2006) postulated that the lower antifungal activity of CTAB could be a result of reversal of fungal cell surface charge and not due to cell lysis, as observed with SDS. Nevertheless, Yu et al. (2015) declared that the surfactants, especially CTAB, showed a strong inhibitory effect on hyphal development and biofilms.

Interestingly, biofilm formation was generally inhibited by *E. faecalis–*CFS compared with the untreated control. This finding is in accordance with other research that shows that *E. faecalis*–CFS is a promising antifungal agent that inhibits morphogenesis and biofilm formation ability of *C. albicans*, as both pathogens inhibit each other’s virulence, promoting a non-pathogenic role in the host (Shekh and Roy, 2012; Cruz et al., 2013; Graham et al., 2017).

To identify the effect of the tested antifungal agents on *C. albicans* morphogenesis, we assessed expression of three hypha-specific genes (*HYR*1, *HWP*1, and *ALS*3) and the hyphal regulator *RAS*1, under hypha-inducing conditions. Transcript analysis showed that expression was downregulated, and therefore, cinnamon oil and SDS followed by CTAB and *E. faecalis*–CFS might exert their anti-biofilm effect *via* perturbation of cell wall integrity and inhibition of adherence. The obtained results confirm the findings of previous studies (Tsang et al., 2012; Inci et al., 2013) that downregulation of adhesion-related genes, *ALS*3 and *HWP*1, reduces biofilm formation by inhibiting adhesion of *C. albicans* cells. The downregulation of the *RAS*1 gene in *C. albicans* biofilm in the presence of the tested antifungal agents, particularly cinnamon oil and SDS, implies that they might inhibit the expression of filament-inducing genes *via* an *RAS*1-mediated way (Ras1-cAMP-Efg1 and Cek1-Cph1p pathways). As a result, hyphal growth and biofilm formation are inhibited (Han et al., 2011).

SEM analysis revealed intact biofilm formation by untreated cells in 48 h, whereas cinnamon treated cells exhibited disorganization of biofilm stages. This appears to be because of the interference of oil with cell membrane integrity, as evidenced by the shrinkage of the cell surface and lysis of sessile cells. Similar observations were reported for *Cymbopogon citrates*, *Syzygium aromaticum*, *Carum copticum* EOs, and *Thymus vulgaris* against *C. albicans* biofilm (Sajjad et al., 2012; Khan et al., 2014). This indicates that *Candida* cells in the biofilm stage cannot gain increased tolerance to test oils as they do against antifungal drugs.

This study is the first to examine the immunopharmacological activities of the different antifungal agents on PBLCs (neutrophils, monocytes, and macrophages) against *C. albicans* biofilms. The results revealed that planktonic cells were easily phagocytized (**Figure 3**), in contrast to *C. albicans* within the biofilm that was resistant to phagocytes. This ease in the cells’ phagocytic capacity toward planktonic cells may be due to the powerful inherent property of PBLCs to engulf particulate pathogens. Other causes may be the induction of cytokines/chemokines secretions that upregulate the cells’ surface makers’ expression and phagocytic cell communication with the host and immune cells; the release of antimicrobial substances that inhibit biofilm formation, such as lactoferrin protein (Velliyagounder et al., 2015) and the release of neutrophil extracellular traps (NETs) (Kim et al., 2005). Among the tested compounds, cinnamon oil followed by *E. faecalis*–CFS was the most effective anti-biofilm agents as they enhanced PBLCs to eradicate and clear the *C. albicans* biofilms after 30–60 min at the preformed and established phases (**Figures 4**, **5**). Enlargement and aggregation of the PBLCs were observed during the different time intervals of cells addition to biofilm indicating cells activation and functional changes (Elmowalid, 2012; Elmowalid et al., 2013). These results suggested that biofilms of *C. albicans* were significantly more susceptible to the cinnamon, *E. faecalis*–CFS, and SDS compounds in comparison with the standard FLC antifungal. These agents may exert their anti-biofilm activity through modulation of the external proteins or architecture of the biofilm that results in loosening the structure and biofilm adherent cell separation; interference with the gene (s) controlling and expressing biofilm; regulation of the cytokines/chemokines secretion by the immune cells involved in the immune cells interaction; or through enhancing the secretion of external biofilm inhibitory factors in the culture media as proposed by Chandra et al. (2007). PBLCs and the tested compounds showed little destructive and inhibitory effect on established and preformed biofilms when added alone (**Figures 4**, **5**). This could be explained by the inability of PBLCs to completely engulf *C. albicans* hyphae and other large or aggregated pathogens. Although the NETs are considered an effective neutrophil response against biofilm in other microbial spp., they are not produced in response to *C. albicans* biofilms (Johnson et al., 2016; Kernien et al., 2017). Previous studies also indicated that *C. albicans* biofilms do not trigger neutrophils or monocytes and macrophages to generate reactive oxygen species (ROS), a signaling that initiates NETs formation (Johnson et al., 2016). These impaired responses have been attributed, in part, to the presence of an extracellular matrix, encasing the *C. albicans* cells or masking of external epitopes preventing *C. albicans* recognition by the phagocytes, and hence evasion of the phagocytic response (Netea et al., 2006; Johnson et al., 2016).

## 5 Conclusions

This is the first study exploring the efficacy of five natural, biological, and chemical compounds together with standard antifungal drug against clinical *C. albicans* isolates from different sources at the planktonic and biofilm phases that could be promising, efficient, and cost-effective drugs for the inhibition of *C. albicans* biofilms. It is also elucidates the additive immunomodulatory activities of cinnamon oil and/or *E. faecalis*–CFS to PBLCs. However, it is worth noting that cinnamon oil and *E*. *faecalis–*CFS showed anti-*Candida* and anti-biofilm activities at a lower concentration as compared with the FLC standard drug. Cinnamon oil and *E. faecalis–*CFS were able to inhibit *C. albicans* yeast–hyphal transition and effectively enhanced the PBLCs to rapidly eradicate the biofilm formation at different developmental phases after treatment with those compounds. Hence, future studies may investigate the efficacy of combinational therapy of those compounds with standard drugs in a clinical trial or with other combinations that could lead to novel drug therapies against biofilm forming *C. albicans* in medical settings.

## Data Availability Statement

The datasets presented in this study can be found in the article and supplementary material.

## Author Contributions

Conceptualization: MH, YT, and GE; methodology: YT, GE, MH, and DA; validation: YT, GE, MH, and DA; formal analysis: YT, GE, MH, DA, AS, and TS; investigation: YT, GE, MH, DA, AS, and TS; data curation: YT, GE, MH, DA, AS, and TS; writing—original draft preparation: YT; writing—review and editing: YT and GE. All authors contributed to the article and approved the submitted version.

## Conflict of Interest

The authors declare that the research was conducted in the absence of any commercial or financial relationships that could be construed as a potential conflict of interest.

## Publisher’s Note

All claims expressed in this article are solely those of the authors and do not necessarily represent those of their affiliated organizations, or those of the publisher, the editors and the reviewers. Any product that may be evaluated in this article, or claim that may be made by its manufacturer, is not guaranteed or endorsed by the publisher.

## Supplementary Material

Supplementary Figure 1 Time-lapse microscopy of *C. albicans* biofilm formation. At 2-h initial adherence of yeast cells followed by germination and micro-colony formation (4 h), filamentation and monolayer development (6 h), proliferation (18 h), and maturation (24–48 h); mature biofilm becomes thick with extracellular polysaccharide layer in which blastoconidia and pseudohyphae are embedded.
