# Supplementary figures and images for: Promising Anti-Biofilm Agents and Phagocytes Enhancers for the Treatment of Candida albicans Biofilm–Associated Infections
Source: Front Cell Infect Microbiol. 2022 Jul 1;12:807218. doi: 10.3389/fcimb.2022.807218 (PMC9283759; doi:10.3389/fcimb.2022.807218)

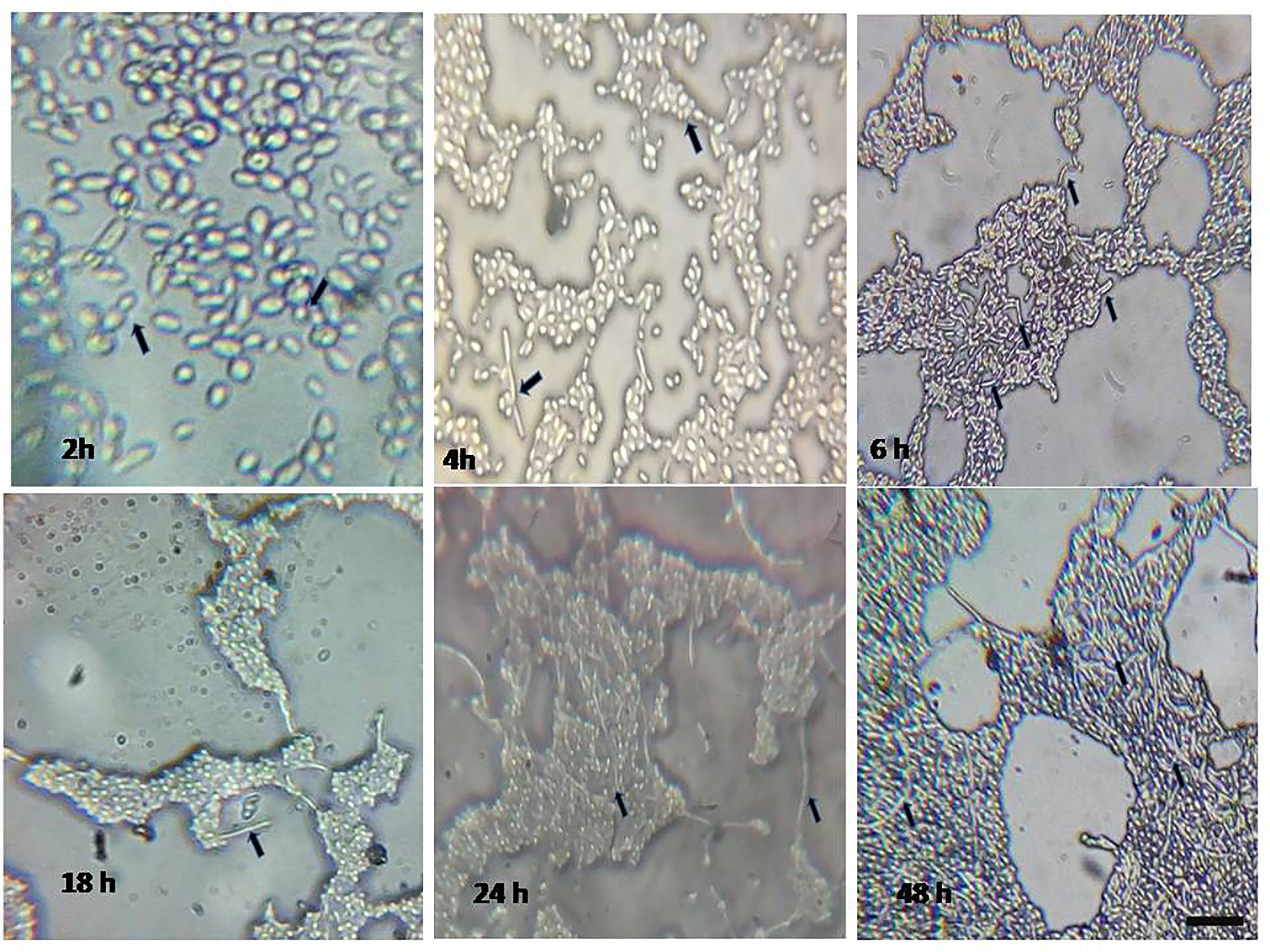

Supplement: Supplementary Figure 1 — Time-lapse microscopy of C. albicans biofilm formation. At 2-h initial adherence of yeast cells followed by germination and micro-colony formation (4 h), filamentation and monolayer development (6 h), proliferation (18 h), and maturation (24–48 h); mature biofilm becomes thick with extracellular polysaccharide layer in which blastoconidia and pseudohyphae are embedded. [file Image_1.jpeg]
